# Responsible artificial intelligence (AI) in healthcare: a paradigm shift in leadership and strategic management

**DOI:** 10.1108/LHS-01-2025-0018

**Published:** 2025-09-09

**Authors:** Amlan Haque

**Affiliations:** School of Business and Law, CQUniversity Sydney, Sydney, Australia

**Keywords:** Responsible AI, Healthcare leadership, Strategic management, AI integration, Healthcare management

## Abstract

**Purpose:**

This paper aims to explore the paradigm shift in leadership and strategic management driven by the integration of responsible artificial intelligence (AI) in healthcare. It explores the evolving role of leadership in adapting to AI technologies while ensuring ethical governance, transparency and accountability in healthcare decision-making.

**Design/methodology/approach:**

This study conducts a comprehensive review of current literature, case studies and industry reports to evaluate the implications of responsible AI adoption in healthcare leadership. It focuses on key areas such as AI-driven decision-making, resource optimisation, crisis management and patient care, while also addressing challenges in integrating AI technologies effectively.

**Findings:**

The integration of AI in healthcare is transforming leadership from traditional, experience-based decision-making to data-driven, AI-enhanced strategies. Responsible leadership emphasises addressing ethical concerns such as bias, transparency and accountability. AI technologies improve resource allocation, crisis management and patient care, but challenges such as workforce resistance and the need for upskilling healthcare professionals remain.

**Practical implications:**

Healthcare leaders must adopt a responsible leadership framework that balances AI’s potential with ethical and human-centred care principles. Recommendations include developing AI literacy programmes for healthcare professionals, ensuring inclusivity in AI algorithms and establishing governance policies that promote transparency and accountability in AI applications.

**Originality/value:**

This paper provides a critical, forward-looking perspective on how responsible AI can drive a paradigm shift in healthcare leadership. It offers novel insights into the integration of AI within healthcare organisations, emphasising the need for leadership that prioritises ethical AI usage and promotes patient well-being in a rapidly evolving digital landscape.

## Introduction

Artificial intelligence (AI) is fundamentally transforming industries across the globe, and healthcare is no exception. In an era where data-driven decision-making is becoming the norm, AI is poised to revolutionise how healthcare systems operate, from enhancing patient care to streamlining administrative processes (Sposato, 2025; Gagliardi and Tomaselli, 2025). Technologies like predictive analytics, robotics and AI-driven automation are at the forefront of this transformation, enabling clinicians to make more informed decisions, improve patient outcomes and optimise resources (Sposato, 2025). Predictive analytics, for example, empowers healthcare providers to identify potential risks early, leading to proactive interventions that can save lives and reduce costs. Similarly, robotics has revolutionised surgeries, offering precision that minimises human error and accelerates recovery times, while AI-powered automation alleviates administrative burdens such as scheduling, billing and data management (Barrett *et al.*, 2019; Ruoxing *et al.*, 2025).

This shift towards AI integration represents not just a technological advancement but a fundamental change in how healthcare is delivered, requiring a rethinking of leadership and strategic management in the sector (Sposato, 2025). Healthcare leaders must now navigate the complexities of AI adoption while ensuring ethical governance, transparency and accountability in decision-making processes. While AI brings immense potential, it also presents significant challenges, such as overcoming workforce resistance and ensuring inclusivity in AI algorithms (Gagliardi and Tomaselli, 2025). Addressing these concerns requires a responsible approach to leadership that prioritises ethical AI usage and aligns technological advancements with the core values of patient-centred care. This paper explores how healthcare leaders can harness AI’s full potential while addressing the risks and challenges associated with its use.

## Methodology

This paper uses a qualitative literature review methodology to critically examine the leadership and strategic management paradigm shift driven by responsible AI integration in healthcare. The review method facilitates synthesising multidisciplinary academic scholarship, policy frameworks and industry reports to comprehensively understand responsible AI adoption, associated ethical challenges and leadership implications within healthcare contexts.

### Information sources and selection criteria

A comprehensive search was conducted across leading academic databases, including Scopus, Web of Science, PubMed and Google Scholar, targeting peer-reviewed journal articles, authoritative policy documents and relevant case studies published between 2018 and 2025 to capture recent developments. Key search terms included “responsible AI”, “AI ethics in healthcare”, “healthcare leadership and AI”, “AI governance”, “ethical AI challenges” and “strategic management of AI in healthcare”. Reports from reputable organisations such as the National Academy of Medicine and the Brookings Institution were also included to provide a robust policy context.

Sources were selected based on their explicit focus on responsible AI integration in healthcare, emphasising ethical governance, leadership roles, strategic management and AI-related risks such as bias, transparency, accountability and workforce impact. Only high-quality, recent publications – peer-reviewed articles, conference papers and official policy reports, mostly from 2018 to 2025 – were included to ensure relevance, credibility and coverage of emerging trends.

## Grounding AI leadership in empirical and policy contexts

A broader body of evidence from scholarly literature, case studies and policy reports is essential to address AI’s ethical and operational dimensions in healthcare. Many studies highlight the need for robust governance, risk and compliance (GRC) frameworks to guide AI integration in clinical settings. For example, a recent industry report emphasises that without clearly defined GRC protocols, AI implementation may lead to unintended consequences, including algorithmic discrimination and liability gaps (Accesswire, 2024). Similarly, Brookings Institution scholars argue that a national framework for responsible AI is crucial to ensure equitable access and mitigate disparities across communities, especially among underserved populations (West and Allen, 2024).

Scholarly discourse further supports the alignment of AI with ethical leadership and corporate social responsibility (Haque, 2021). Gagliardi and Tomaselli (2025) emphasise that AI adoption post-COVID-19 must be linked with social accountability to ensure public trust and sustainability in healthcare. Ethical leadership has also been reimagined through emerging paradigms such as fractal AI, which enables more adaptive and inclusive decision-making in public health systems (Chen and Ryoo, 2025). In parallel, the National Academy of Medicine (2024) has proposed a comprehensive “AI Code of Conduct” for healthcare, underscoring transparency, inclusivity and accountability as foundational principles for AI deployment in clinical practice.

Operationally, AI is transforming healthcare workforce management by enabling predictive scheduling, talent optimisation and digital workflow enhancements (Saeed *et al.*, 2025). As AI assumes greater responsibility in administrative and clinical processes, strategic management approaches are needed to bridge technology and human expertise. Ruoxing *et al.* (2025) demonstrate how artificial neural networks can be leveraged to optimise human resource allocation while preserving strategic oversight. These insights converge on the need for leadership that is not only technologically competent but also ethically anchored.

Together, these scholarly and industry sources underscore the critical role of responsible leadership in ensuring that AI in healthcare is efficient, equitable, transparent and aligned with the public interest.

## Significance of responsible AI

As AI’s role in healthcare deepens, the concept of responsible AI takes centre stage. Responsible AI entails the ethical use of AI technologies, ensuring they are applied transparently, equitably and safely. In healthcare leadership, responsible AI is essential for guiding decisions affecting patient care and organisational strategies. However, challenges abound. AI systems can perpetuate biases, raising concerns over fairness, accountability and transparency if not carefully managed. Data privacy and the explainability of AI-driven decisions are crucial in maintaining public trust and safeguarding patient interests (Kumar *et al.*, 2023). Responsible leadership in this context means deploying AI effectively and ensuring that AI applications are continuously monitored for ethical compliance and equity, preventing harm and ensuring that vulnerable groups are not disadvantaged (Gagliardi and Tomaselli, 2025). The following sections explore the evolving role of AI in healthcare leadership, focusing on the ethical and operational challenges accompanying its implementation. It analyses how responsible AI can address these challenges and ensure effective leadership within the healthcare system. Specifically, the research aims to address the following key questions:

*Q1*. How is AI transforming leadership and strategic management in healthcare?

AI is reshaping healthcare leadership by providing data-driven insights that facilitate strategic decision-making. From improving resource allocation to enhancing patient outcomes through predictive models, AI is helping healthcare leaders optimise organisational strategies and patient care (Subrahmanyam, 2025). The shift to AI-enabled leadership necessitates rethinking traditional leadership approaches and encourages a more data-centric, evidence-based model of healthcare management (Ennis-O’Connor and O’Connor, 2024):

*Q2*. What are the ethical challenges and operational risks associated with AI implementation?

AI’s integration into healthcare presents several ethical dilemmas, including concerns about algorithmic bias, privacy violations, and transparency in decision-making. These issues raise questions about how AI systems are designed and deployed and whether they might inadvertently perpetuate inequalities in care (Yadav *et al.*, 2024). Operational risks such as data security vulnerabilities, the potential for over-reliance on technology and challenges in workforce adaptation also need careful consideration (Movahed and Bilderback, 2024):

*Q3*. How can responsible AI ensure effective leadership and accountability in healthcare?

Responsible AI practices ensure healthcare leadership remains accountable, ethical and aligned with patient-centred goals. By incorporating ethical principles into AI design, such as fairness, explainability and transparency, healthcare leaders can mitigate risks and foster a more inclusive approach to care. Moreover, continuous evaluation of AI systems for biases and their real-world implications is essential in maintaining leadership accountability and public trust (Fernandes *et al.*, 2024; Kumar *et al.*, 2023). Integrating AI into healthcare management is undoubtedly transformative, but its success hinges on adopting a responsible approach that prioritises ethical leadership and patient welfare.

## AI and the evolution of healthcare leadership

The evolution of healthcare leadership is experiencing a profound transformation as AI becomes a central tool in decision-making and strategic management. Historically, healthcare leadership has been guided by intuition, clinical expertise and experience. However, as healthcare systems increasingly harness vast quantities of data for decision-making, leadership models are shifting towards data-driven approaches enabled by AI technologies. This shift redefines leadership roles, particularly in areas like resource optimisation, crisis management and patient care, aligning leadership with the demands of modern healthcare systems. AI is moving from reactive, experience-based decision-making to proactive, data-informed strategies. Healthcare leaders now face the challenge of integrating AI while ensuring leadership remains grounded in human-centred care, ethical responsibility and accountability.

As AI advances, healthcare leaders must adapt to a new paradigm that emphasises responsible, transparent and accountable leadership. Responsible AI leadership goes beyond merely adopting AI technologies; it requires a framework that integrates ethical considerations, inclusivity and transparency. This framework ensures that AI-driven strategies contribute to better healthcare outcomes while mitigating risks. For instance, AI leadership must address the ethical challenges related to data privacy, algorithmic bias and equity in healthcare delivery. AI is not just a tool for efficiency but must be used responsibly to reflect the diverse social, cultural and ethical contexts within which healthcare operates (Gagliardi and Tomaselli, 2025; Ruoxing *et al.*, 2025). Leaders must balance the operational goals of AI deployment with the societal values of fairness, inclusivity and patient autonomy.

Strategically, AI has proven to be a powerful ally in resource management, crisis response and workforce planning. A compelling example of AI’s role in leadership can be seen in its application during high-pressure situations, such as the COVID-19 pandemic. AI-powered systems, including predictive analytics and machine learning algorithms, have enabled healthcare leaders to optimise resource allocation, forecast patient demand and manage hospital capacities more effectively. During the pandemic, AI tools were used to predict surges in patient numbers, direct resources to areas of greatest need and streamline the triage process, contributing to improved hospital responses under pressure (Subrahmanyam, 2025). These AI-driven systems enable healthcare leaders to make better-informed decisions, reduce response times and enhance the quality of care during crises. Moreover, AI transforms patient-centred leadership, significantly impacting how healthcare systems engage with patients. AI tools promote personalised care, empower patients to manage chronic conditions and facilitate self-care, improving health outcomes and patient satisfaction (Barrett *et al.*, 2019). However, integrating AI into patient care raises important challenges in managing patient expectations. As AI becomes more embedded in healthcare, patients may have concerns about the role of machines in their healthcare decisions. To foster trust in AI technologies, healthcare leaders must ensure transparency in AI interventions, addressing concerns regarding AI’s limitations and ethical implications in medical decision-making. Educating patients about AI’s capabilities and ensuring that these interventions align with the values of patient autonomy is essential for maintaining patient trust.

The evolution of healthcare leadership in the AI era presents unprecedented opportunities and significant challenges. Responsible leadership becomes more critical as AI continues to reshape strategic management and patient care. Healthcare leaders must embrace AI’s potential to optimise healthcare systems while ensuring that AI’s integration is guided by ethical principles prioritising transparency, patient well-being and accountability (Haque, 2021). Through a responsible leadership model, AI can drive improved healthcare outcomes, but only if managed with the highest ethical and social responsibility standards.

## Key challenges for responsible AI-driven healthcare leadership

As AI continues to gain traction in healthcare, healthcare leaders must confront several key challenges to ensure that AI-driven innovations are both responsible and effective. These challenges range from ethical dilemmas to organisational obstacles, requiring careful consideration and strategic planning to navigate effectively.

### Ethical and responsible AI dilemmas

One of the most significant challenges in deploying AI in healthcare is ensuring fairness and mitigating bias in AI systems. AI algorithms often rely on large data sets, which, if not representative of diverse populations, can result in biased outcomes. For instance, biases in training data related to race, gender or socio-economic status can lead to AI systems that disproportionately benefit certain groups over others, thus exacerbating healthcare inequalities (Kumar, Dwivedi, and Anand, 2023). Healthcare leaders must prioritise identifying and mitigating biases in AI models, ensuring that these technologies support equitable healthcare delivery.

In AI-led decisions, transparency, accountability and trustworthiness also present critical ethical concerns. Many AI systems operate as “black boxes”, where the decision-making process is not easily understood or explainable. This lack of transparency can erode trust among patients and healthcare professionals, mainly when AI makes critical patient care decisions. Leaders must advocate for developing and using explainable AI models to improve the trustworthiness and accountability of AI-driven healthcare decisions (Ruoxing *et al.*, 2025). This transparency ensures that decisions can be scrutinised and adjusted when necessary, promoting ethical practice.

Furthermore, data security and patient privacy are paramount in AI applications. AI systems often require access to vast amounts of sensitive patient data to function effectively, raising concerns over data breaches, unauthorised access and misuse. To safeguard patient data, leaders must establish robust cybersecurity measures and ensure compliance with regulations such as the Health Insurance Portability and Accountability Act. Maintaining privacy while utilising AI for more personalised and efficient healthcare is one of the most pressing challenges in modern healthcare leadership (Gagliardi and Tomaselli, 2025).

### Organisational and workforce challenges

In addition to ethical concerns, healthcare organisations face significant internal challenges in adopting AI technologies. A notable barrier is healthcare professionals’ resistance to AI adoption. Many healthcare workers are apprehensive about AI systems replacing their roles or undermining their expertise. This resistance can be extreme in clinical settings, where professionals may perceive AI as threatening their autonomy or professional judgment. Healthcare leaders must foster a culture of collaboration and transparency, ensuring that AI is seen as a tool to augment, rather than replace, human expertise (Subrahmanyam, 2025). Encouraging open dialogue and demonstrating the potential benefits of AI can help reduce resistance and promote a more welcoming attitude towards innovation.

Another challenge is the need to upskill leaders and clinicians in AI literacy to facilitate responsible adoption. With AI becoming integral to healthcare decision-making, healthcare professionals must have the knowledge and skills to interact effectively with these systems (Kumar *et al.*, 2023; Gagliardi and Tomaselli, 2025). This includes understanding the technical aspects of AI and grasping its ethical implications. Leaders should prioritise AI education and training initiatives to empower clinicians and administrators to make informed decisions about AI use (Movahed and Bilderback, 2024). By fostering AI literacy across the workforce, healthcare organisations can better integrate AI responsibly and effectively.

Finally, balancing automation with human expertise is a critical issue. While AI can enhance efficiency and improve patient outcomes, it cannot replace the nuanced judgment and empathy that human clinicians provide. Ensuring human oversight in AI-driven healthcare systems balances technology and human care (Sposato, 2025; Gagliardi and Tomaselli, 2025). Healthcare leaders must design systems where AI tools complement, rather than supplant, human expertise. This integration requires carefully calibrating AI’s capabilities, focusing on maintaining the centrality of human decision-making in complex or sensitive situations.

## Responsible leadership framework for AI integration

As AI continues to reshape the healthcare landscape, leadership’s role in ensuring responsible AI integration becomes paramount. A responsible leadership framework for AI requires the adoption of core principles focused on ethical governance, inclusivity and transparency, as well as developing best practices that promote collaboration between humans and AI (Kumar *et al.*, 2023).

### Core principles of responsible AI leadership

One of the key principles of responsible AI leadership is ensuring robust ethical governance. Healthcare leaders must be central in maintaining transparency and accountability in AI deployment. Ethical AI governance involves the creation of clear guidelines that prioritise patient safety, data privacy and the equitable application of AI technologies (Kumar *et al.*, 2023). Leaders must oversee the development of AI strategies that optimise operational efficiency and align with moral and social imperatives. For example, leadership can advocate for regular audits of AI systems to ensure that decisions made by these technologies remain transparent and explainable, which fosters trust among patients and healthcare professionals alike (Kumar *et al.*, 2023; Gagliardi and Tomaselli, 2025).

Another essential component is inclusivity in AI algorithms. To prevent discrimination and bias in AI-driven decision-making, it is vital to ensure that AI systems are built using diverse and representative data sets. Healthcare leaders must prioritise the inclusion of various patient demographics in AI training data to mitigate the risks of biases that could affect patient care and outcomes (Ruoxing *et al.*, 2025). This approach supports equitable healthcare delivery and enhances the overall efficacy of AI systems by ensuring that they cater to the population’s diverse needs.

### Best practices for integrating AI in leadership

To successfully integrate AI into healthcare systems, leaders must establish AI literacy programmes and provide training to healthcare professionals. By upskilling clinicians and administrative leaders in AI technologies, organisations can better understand how AI can complement their decision-making and enhance patient care (Kumar *et al.*, 2023; Gagliardi and Tomaselli, 2025). AI literacy programmes also help overcome resistance from healthcare professionals who may fear that automation will replace human expertise. Instead, these programmes should emphasise that AI is a tool to augment human capabilities, improving the quality of care while respecting the role of healthcare providers (Sposato, 2025). These initiatives are integral to cultivating an AI-ready workforce that is confident and responsible in using AI technologies.

Furthermore, healthcare leaders must establish frameworks for AI-human collaboration. Rather than viewing AI as a replacement for human expertise, leaders should promote systems where AI assists healthcare professionals in making informed, data-driven decisions. These collaboration frameworks can include AI tools that provide real-time decision support, helping clinicians make more accurate diagnoses or recommend personalised treatments while leaving critical decision-making to human expertise. This approach improves patient outcomes and strengthens the relationship between healthcare workers and the technologies they use (Fernandes *et al.*, 2024).

Finally, policy recommendations play an essential role in responsible AI integration. Healthcare leaders must advocate for policies that establish ethical standards for AI in healthcare. These policies should address the full spectrum of AI’s impact, from patient privacy concerns to regulating AI-driven decision-making processes (Kumar *et al.*, 2023; Gagliardi and Tomaselli, 2025). Effective governance frameworks should be developed to ensure ongoing accountability, transparency and fairness when deploying AI. Collaboration between healthcare institutions, policymakers and technology developers is necessary to create comprehensive policies that align with ethical standards and the ever-evolving landscape of AI (Yadav *et al.*, 2024).

In light of the preceding conceptual analysis, this paper proposes a structured framework to clarify the essential components of responsible leadership in integrating AI within healthcare systems. While the narrative has explored various dimensions – from ethical governance to workforce adaptation – these insights can be communicated more effectively through a visual representation. Including a conceptual model helps consolidate the discussion and offers a more accessible and actionable guide for healthcare leaders navigating AI adoption.

**Figure 1.**
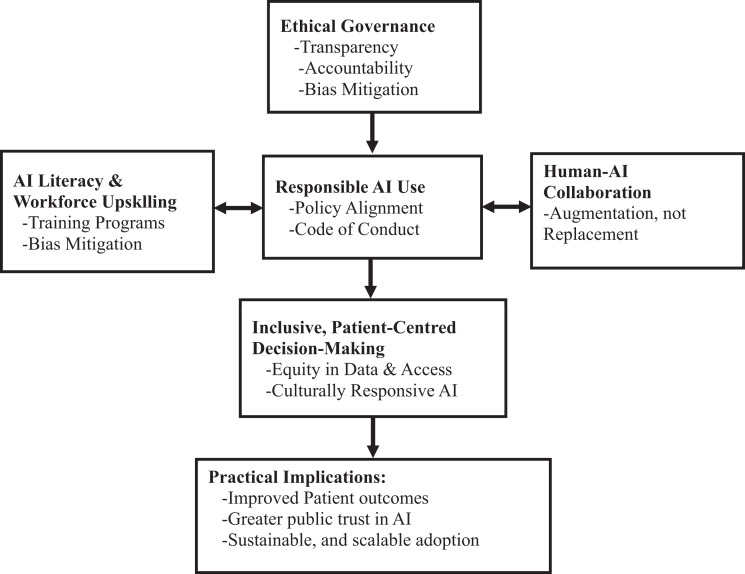
Responsible leadership model for AI integration in healthcare **Source:** Author’s own work

Figure 1 presents the *Responsible Leadership Model for AI Integration in Healthcare*, which captures four interconnected pillars: ethical governance, AI literacy and workforce upskilling human-AI collaboration, and inclusive, patient-centred decision-making. These components form a leadership strategy that balances technological advancement with ethical responsibility and operational readiness.

The model illustrates how transparent and accountable governance structures are the foundation for responsible AI leadership. Simultaneously, AI literacy and human-AI collaboration ensure that AI tools are used to augment – not replace – clinical expertise. Finally, inclusive decision-making processes ensure that AI applications reflect diverse patient needs and support equitable care delivery. This framework integrates insights from recent literature (e.g. Gagliardi and Tomaselli, 2025; Kumar *et al.*, 2023) and recent policy guidance (National Academy of Medicine, 2024), offering a roadmap for implementing AI in ways that align with ethical, strategic and human-centred healthcare values.

## Potential research gaps and corresponding research questions

The growing integration of AI in healthcare has prompted considerable academic and industry attention, particularly regarding its technical capabilities, such as diagnostic accuracy, clinical support and operational efficiency. However, the prevailing body of literature tends to adopt a techno-centric lens, often marginalising the strategic leadership and governance dimensions essential for the ethical and sustainable implementation of AI technologies in complex healthcare settings.

Although some conceptual discussions have begun to address responsible AI, they typically focus on algorithmic fairness, bias mitigation or data security – while underexposing the leadership roles, decision-making adaptations and strategic transformation processes required to embed responsible AI principles into healthcare organisations. Specifically, current research lacks a systematic evaluation of how leaders manage ethical dilemmas, address staff readiness, foster patient trust and ensure accountability in AI-driven environments. There is also a notable absence of an integrated framework that links responsible AI practices with leadership competencies and strategic imperatives.

This review synthesises multidisciplinary insights from healthcare leadership, AI ethic and strategic management to address this significant gap. The following research questions (RQs) guide it:

*RQ1*. How is responsible AI adoption reshaping healthcare organisations’ leadership roles and decision-making processes?

*RQ2*. What are the key ethical and strategic challenges healthcare leaders face when integrating AI technologies?

*RQ3*. How can leadership frameworks be adapted to promote transparency, accountability and human-centred values in AI implementation?

*RQ4*. What strategic competencies and organisational changes are necessary for healthcare leaders to ensure responsible and sustainable AI adoption?

By engaging with these questions, this review contributes to the scholarly discourse on responsible AI by uncovering underexplored leadership dynamics and providing a foundation for future empirical investigations and conceptual framework development. It also offers practical insights for healthcare executives seeking to lead AI-enabled transformations that are both ethically grounded and strategically sound.

## Implication and future directions

This review set out to address a critical gap in the literature: the limited integration of strategic leadership, governance and ethical imperatives in discussions on AI adoption in healthcare. While technological applications of AI – such as diagnostic precision and operational efficiency – are widely covered, the strategic roles of healthcare leaders in ensuring responsible, transparent and human-centred AI implementation remain underexplored.

Guided by four research questions, this review synthesised multidisciplinary insights on responsible AI, ethical leadership and organisational transformation. It highlighted that AI is not merely a technical tool but a strategic catalyst reshaping leadership decision-making, organisational readiness and patient engagement. Responsible AI adoption demands leadership that proactively governs ethical risks, ensures equitable access and aligns AI applications with core healthcare values.

### The future of responsible AI in healthcare leadership

Emerging AI innovations – from deep learning in diagnostics to robotic-assisted surgery – offer transformative potential (Kumar *et al.*, 2023; Gagliardi and Tomaselli, 2025). However, these advancements depend on high-quality, representative data and leadership oversight to mitigate risks like algorithmic bias and loss of human agency (Brookings Institution, 2024; Chen and Ryoo, 2025).

Strategic healthcare leaders must guide their organisations through the ethical, operational and cultural transitions required for responsible AI integration. This includes investing in workforce upskilling, developing AI literacy and fostering effective human-AI collaboration (Movahed and Bilderback, 2024; Yadav *et al.*, 2024).

Table 1 below synthesises the core leadership dimensions from this review, mapped explicitly to the guiding research questions. It is a conceptual scaffold for understanding how leadership must evolve to support responsible AI adoption across five critical pillars:

**Table 1.** Key dimensions of responsible AI leadership and their linkages to research questions in healthcare

Dimension	Description	Supporting references	Related research question(s)
Ethical governance and accountability	Establishing clear policies, codes of conduct and ethical frameworks to ensure responsible AI use and data privacy	Gagliardi and Tomaselli (2025); National Academy of Medicine (2024); Kumar *et al.* (2023); Barrett *et al.* (2019)	RQ2, RQ3
Patient-centred care and engagement	Using AI to enhance personalised, predictive and preventive care while actively involving patients in decision-making	Barrett *et al.* (2019); Ennis-O’Connor and O’Connor (2024); Kumar *et al.* (2023); Fernandes *et al.* (2024)	RQ1, RQ3
Workforce readiness and digital literacy	Preparing healthcare leaders and staff through upskilling education, and building AI literacy for effective adoption	Movahed and Bilderback (2024); Sposato (2025); Subrahmanyam (2025)	RQ4
Human–AI collaboration and decision support	Leveraging AI to augment rather than replace human judgment, promoting ethical and effective joint decision-making	Yadav *et al.* (2024); Subrahmanyam (2025); Ruoxing *et al.* (2025); Barrett *et al.* (2019)	RQ1, RQ2
Sustainability and inclusivity	Ensuring AI applications promote sustainable healthcare outcomes and equitable access across diverse populations	Haque, 2021; Fernandes *et al.* (2024); Gagliardi and Tomaselli (2025); Kumar *et al.* (2023)	RQ2, RQ4

**Source(s):** Author’s own work

1. governance;
2. patient-centred care;
3. workforce development;
4. ethical decision support; and
5. inclusivity.

This table consolidates key findings across disciplines and clarifies how each dimension aligns with the research questions driving this review. It provides a thematic foundation for theory development and strategic practice in healthcare AI leadership.

Future studies should build on this review by, first, empirically testing leadership models that integrate ethical AI governance across varied healthcare settings. Second, researchers should explore sectoral parallels in finance, education and public services to develop cross-sectoral frameworks for responsible AI adoption (West and Allen, 2024). Third, longitudinal evaluations are needed to assess the impact of responsible AI leadership practices on patient trust, equity and system efficiency. Finally, future work should focus on designing visual frameworks or maturity models that healthcare leaders can use to guide responsible AI strategy, training and implementation.

Here, Table 1 is significant as it synthesises critical dimensions of responsible AI integration in healthcare leadership and strategic management, drawing from recent multidisciplinary research. It highlights key pillars such as ethical governance (Gagliardi and Tomaselli, 2025; Kumar *et al.*, 2023), AI literacy and workforce readiness (Movahed and Bilderback, 2024; Sposato, 2025), patient-centred care advancements (Barrett *et al.*, 2019; Ennis-O’Connor and O’Connor, 2024) and strategic resource optimisation (Ruoxing *et al.*, 2025). By consolidating these elements into a comprehensive framework, the table provides a clear basis for understanding how responsible AI reshapes leadership roles, emphasising transparency, inclusivity and sustainability (Fernandes *et al.*, 2024; Yadav *et al.*, 2024). This integrated overview supports identifying practical leadership strategies and future research directions in the evolving healthcare AI landscape.

Healthcare leadership must embrace continuous learning and adaptability, fostering a culture that balances innovation with ethical governance and patient rights (Chen and Ryoo, 2025; Kumar *et al.*, 2023). Future research should explore the longitudinal impacts of AI governance frameworks across healthcare and service sectors to refine leadership models further. By bridging lessons from general service industries with healthcare’s unique ethical landscape, research can develop more robust, scalable approaches to responsible AI adoption.

In conclusion, responsible AI leadership in healthcare demands a multidisciplinary, evidence-based approach that anticipates evolving risks and embeds ethical principles at every stage of AI integration. This holistic strategy will enable healthcare systems to leverage AI’s transformative potential and maintain public trust and equitable patient outcomes in an increasingly digital future.

## Conclusion

Effective and ethical leadership is the linchpin for responsible AI integration in healthcare. While AI promises to revolutionise healthcare by enhancing efficiency, precision and personalisation (Barrett *et al.*, 2019; Kumar *et al.*, 2023), its true potential hinges on a human-centred vision that prioritises ethical oversight, transparency and inclusivity (Fernandes *et al.*, 2024; Gagliardi and Tomaselli, 2025). Healthcare leaders must ensure that AI functions as a tool to augment, rather than replace, human expertise, fostering collaboration between technology and professionals to improve patient outcomes and optimise resources (Yadav *et al.*, 2024; Ruoxing *et al.*, 2025).

As AI capabilities advance, leadership frameworks require strategic agility and robust governance structures to embed accountability, fairness and ethical rigour at the heart of digital transformation (Chen and Ryoo, 2025; National Academy of Medicine, 2024). Such leadership is critical for upholding core healthcare values and promoting equitable access to care across diverse populations (Fernandes *et al.*, 2024; Ennis-O’Connor and O’Connor, 2024).

This review foregrounds leadership as the essential strategic enabler of responsible, sustainable AI adoption in healthcare. It lays the groundwork for future conceptual, empirical and policy research that guides healthcare systems in navigating AI’s vast promise and risks with foresight, integrity and a commitment to human dignity (Sposato, 2025; West and Allen, 2024). Ultimately, realising AI’s transformative potential depends on leaders dedicated to responsible innovation – balancing cutting-edge technology with the enduring human elements of care.
